# Molecular detection and characterisation of *Mycoplasma* species in community owned dogs of Kerala, a South Indian State

**DOI:** 10.17221/116/2023-VETMED

**Published:** 2024-09-29

**Authors:** Gatchanda Shravan Kumar, Anju Varghese, Prabodh Kumar Hembram, Karapparambu Gopalan Ajith Kumar, Chundayil Kalarickal Deepa, Able Varghese, Reghu Ravindran

**Affiliations:** ^1^Department of Veterinary Parasitology, College of Veterinary and Animal Sciences, Pookode, Kerala Veterinary and Animal Sciences University, Pookode, Wayanad, Kerala, India; ^2^Veterinary Consultant, Zootopia Veterinary Clinic, Chalakudy, Kerala, India

**Keywords:** *16S rRNA*, dogs, haplotype networks, *Mycoplasma haemocanis*, phylogenetic analysis

## Abstract

Haemotropic mycoplasmas (haemoplasmas) are obligate epierythrocytic bacteria that infect a wide range of vertebrate hosts. The molecular characterisation of *Mycoplasma* organisms in dogs has not previously been attempted from India. Hence, in the present study, the molecular characterisation of *Mycoplasma* spp. in dogs of different zones of Kerala was attempted using *16S rRNA* gene. An overall prevalence of 18% for *M. haemocanis* was detected. The NCBI-BLAST analysis of all the selected sequences revealed > 99% identity with the sequences of *M. haemocanis.* The phylogenetic analysis revealed clustering of *M. haemocanis* and *M. haemofelis* in a single clade indicating low genetic variability. It was further supported by the genetic distance data and haplotype analysis.

Haemotropic mycoplasmas (haemoplasmas) are obligate epierythrocytic bacteria without a cell wall that infect a wide range of vertebrate hosts. Canine haemotropic *Mycoplasma* infections are usually chronic and subclinical. However, in splenectomised or immunosuppressed animals and in animals with concurrent infections, it can result in acute cases leading to haemolytic anaemia (Messick 2004). The pathogen adheres to the erythrocytes of the vertebrate hosts and the adherence mechanisms appear to be related to the production of a dint in the red blood cell membrane at the point of attachment (Ashford 2001).

*Mycoplasma haemocanis* and *Candidatus* Myco-plasma haematoparvum were the two most common species of *Mycoplasma* previously reported throughout the world in dogs (Cortese et al. 2020). Other haemoplasma species reported in dogs include *Candidatus* Mycoplasma haemominutum (feline haemoplasma species) from China and Japan (Zhuang et al. 2009; Obara et al. 2011*)* and *Candidatus* Mycoplasma haemobos (Barker et al. 2012; Hii et al. 2012). The prevalence of canine haemoplasmas might change depending on the climatic conditions in the sampling areas, the presence and distribution of vector species and the host involved (community owned or owned dogs) (Baker and Tasker 2016; Guo et al. 2017; Aktas and Ozubek 2018; Maggi and Kramer 2019). The total dog population reported in India is 9.43 million. In Kerala, 0.84 million dogs exist, out of which 0.3 million are community owned (stray dogs) (Ministry of Agriculture and Department of Animal Husbandry, Dairying and Fisheries 2019). Most of these community owned dogs suffer from malnutrition, diseases and lack of basic health care like deworming, anti-parasitic treatments or vaccination (Massei et al. 2017).

Since *Mycoplasma* spp. are difficult to culture and there is difficulty in conducting experimental studies for assessing the mechanisms of infection, the modes of transmission of the pathogen are not entirely clear. The modes of transmission include the bite of the tick vector *Rhipicephalus sanguineus* (Messick 2004; Baker and Tasker 2016; Aktas and Ozubek 2017) and mechanical means like blood transfusion, fresh blood-contaminated fomites or by blood-sucking arthropods (Messick 2004; Willi et al. 2010; Baker and Tasker 2016). Other routes of transmission include biting and fighting habits in dogs (Sasaki et al. 2008; Willi et al. 2010) and transplacental transmission (Lashnits et al. 2019).

Canine haemoplasmas have not been successfully grown in *in vitro* culture (Roblejo-Arias et al. 2022). Light microscopy provides a preliminary diagnosis based on the cytological identification of the haemoplasma organisms in Giemsa-stained peripheral blood smears (Willi et al. 2010). However, microscopy cannot distinguish between the haemoplasma species from the background debris or stain artifacts (Raimundo et al. 2016). Serological assays are not yet routinely available (Cortese et al. 2020). Polymerase chain reaction (PCR), both conventional and quantitative, is considered the gold standard for the detection and differentiation of species of haemoplasmas (Wengi et al. 2008; Gentilini et al. 2009; Cortese et al. 2020). The *16S rRNA* gene is the most widely used molecular marker in the PCR-based assays (Willi et al. 2010) as this gene is highly conserved in this bacterium (Clarridge 2004).

Data regarding the prevalence of haemoplasma infection in dogs in India remain scarce. There is one report of a *Mycoplasma* infection in dogs from north India (Abd Rani et al. 2011) where the pathogen was detected by PCR and the other report was from south India where the organism was identified morphologically (Preena et al. 2020). Recently, a phylogenetic analysis of feline *Mycoplasma* species was performed in domestic cats of Kerala (Malangmei et al. 2021). As the canine *Mycoplasma* has not been characterised from India, the present study was carried out.

## MATERIAL AND METHODS

### Ethical statement

All the study procedures and protocols were approved by the institutional animal ethics committee (IAEC) of the College of Veterinary and Animal Sciences, Pookode, Kerala which follows the guidelines prescribed by the committee for the purpose of control and supervision of experiments on animals (CPCSEA) under the Ministry of Fisheries, Animal Husbandry and Dairying, Government of India. The committee did not deem it necessary for this research group to obtain formal approval to conduct this study, since the collection of samples from the dogs were performed for the disease diagnosis.

### Animals and samples

A total of 150 community owned, apparently healthy, dogs from north (Kozhikode, “*n* = 50”), central (Thrissur, “*n* = 34” and Ernakulam, “*n* = 16”) and south (Thiruvananthapuram, “*n* = 50”) Kerala were screened for infections with the *Mycoplasma* species from May 2020 to May 2021. Whole blood samples in ethylenediaminetetraacetic acid (EDTA) were collected and thin peripheral blood smears were also prepared from these dogs. The blood smears were stained with the Giemsa stain and examined for *Mycoplasma* organisms under the oil immersion (100× magnification) objective of a compound microscope (Leica DM 1000; Leica Microsystems GmbH, Wetzlar, Germany).

### Polymerase chain reaction

Genomic DNA was isolated from the whole blood samples using a DNeasy blood and tissue kit (Qiagen, Hilden, Germany) according to the manufacturer’s protocol. The extracted DNA was eluted in 100 μl of a DNA elution buffer and stored at –20 °C. The polymerase chain reaction (PCR) was carried out in a final reaction volume of 25 μl containing 0.25 Mm dNTPs, 1 U DyNAzyme II DNA polymerase (Thermo Scientific, Waltham, USA), 50–100 ng of the template DNA and 10 pmol each of the forward and reverse primers in a thermal cycler (Eppendorf, Hamburg, Germany). The primers used in the study for the amplification of *16S rRNA* gene (595 bp) of *Mycoplasma* spp. were (HBT-F: 5' ATACGGCCCATATTCCTACG 3'; HBT-R: 5' TGCTCCACCACTTGTTCA 3') (Criado-Fornelio et al. 2003). The cycling conditions included an initial denaturation at 94 °C for 10 min, followed by 40 cycles of denaturation at 95 °C for 30 s, annealing at 60 °C for 30 s and extension at 72 °C for 30 seconds. A final extension was performed at 72 °C for 10 minutes. The amplified PCR products were run on two percent agarose gel containing ethidium bromide (0.5 μg/ml) and visualised under ultraviolet (UV) light in a gel documentation system (Uvitech, Cambridge, UK).

### Sequence analysis

The amplified PCR products were purified using NucleoSpin^®^ Gel and PCR Clean-up kits (Macherey-Nagel, Düren, Germany) as per the manufacturer’s protocols. The purified PCR products were custom sequenced at AgriGenome Labs Private Ltd., Cochin, Kerala by Sanger’s dideoxy chain termination method. The nucleotide sequences were analysed for their identity using NCBI-BLAST (www.ncbi.nlm.nih.gov/BLAST) and submitted to GenBank.

### Phylogenetic analysis

The partial nucleotide sequences of the *16S rRNA* gene of *Mycoplasma* isolates from Kerala, generated in the present study, were aligned with the previously published sequences of *Mycoplasma* spp. available in the GenBank using the Clustal W program (MEGA v11.0) with suitable models. The evolutionary history of *Mycoplasma* isolates based on the *16S rRNA* gene was inferred by the Maximum Likelihood method based on the Kimura-2-Parameter with Gamma distribution with 43 nucleotide sequences (Table 1). The reliability of the topologies was tested by bootstrapping with 1 000 replications. The mean group genetic distances of *M. haemocanis and M. haemofelis* were also calculated using the Kimura-2-Parameter model for the *16S rDNA* gene.

**Table 1:** The sequences used for the phylogenetic and haplotype analysis

Phylogenetic analysis	Haplotype analysis
*M. haemocanis*: AF197337 (USA), KU765208 (Thailand), AY529641 (Japan), KP715860 (Brazil), GQ129115 (Italy), MZ221167 (Cuba), MN294708 (Mexico), MT345534 (South Korea)	*M. haemocanis*: AB848714 AY529641 (Japan), KP715857, KP715858, KP715859, KP715860 (Nigeria), GQ129115, GQ129116, GQ129117, GQ129118, GQ129119 (Italy), MZ221167, MZ221166, MZ22118, MZ221169, MZ221170, MZ221171, MZ221172, MZ221173, MZ221174 (Cuba), KY368749, MG594501, MG594502 (Turkey), KY117654, KY117656, KY117658, KY117659 (Chile), MW633326 (Angola), MN294708 (Mexico), MT345534 (South Korea), EU442623 (Brazil), MW784616, MW784617, MW784618 (Iraq), EF416566, EF416567, EF416568 (Switzerland), AF197337 (USA), KU765208, KJ858513 (Taiwan), KT359591 (Thailand)
Uncultured *Mycoplasma* sp. clone: KY863524 (Brazil), KX519722 (China) *M. haemofelis:* AY150984 (UK), AY150977 (Australia), EU839978 (Italy), AF178677(USA), AY150972 (France), MN2400855 (Kerala, India) *Candidatus* Mycoplasma haematoparvum: AY 532390 (UK), MZ221175 (Cuba) *Candidatus* Mycoplasma haemominutum: U88564 (USA), AY150974 (Israel) *Candidatus* Mycoplasma haemolamae: MN640395, MN640397 (Chile) *Mycoplasma suis:* FN984917 (Switzerland), FN436019 (Germany) *Mycoplasma ovis*: AF338268 (Germany), MF377463 (Turkey), *Mycoplasma wenyonii*: MG948627, MG948625 (Cuba), *M. hominis*: M24473, NR118700 (USA)	

Haplotype analyses by PopART v1.7 and dnaSP v6.12.03 were performed for the establishment of the evolutionary relationship and genetic diversity among the Indian isolates of *M. haemocanis* (Bandelt et al. 1999; Librado and Rozas 2009). A total of 53 sequences of *M. haemocanis* isolates from different countries were used for the haplotype analysis (Table 1).

## RESULTS

**Figure 1:**
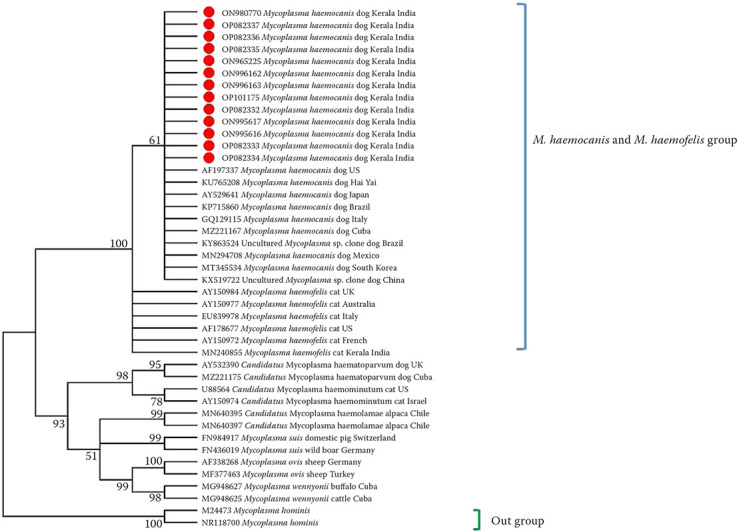
Phylogenetic tree of *M. haemocanis* using the *16S rDNA* sequences generated from 13 isolates from Kerala with a total of 43 sequences available in the database

The microscopy could not detect *Mycoplasma* organisms in any of the examined blood smears. The genus specific primers targeting the *16S rRNA* gene of *Mycoplasma* amplified a 595 bp fragment in 28/150 (18.6%) of the samples. The zone-wise study showed that 18% (9/50) of the dogs from north zone (Kozikode), 20% (10/50) from the central zone (6/34 from Thrissur and 4/16 from Ernakulam) and 18% (9/50) from the south zone (Thiruvanathapuram) were positive for *Mycoplasma* infection. The nucleotide sequences generated in the present study were submitted to the GenBank (GenBank Accession Nos.: ON965225, ON980770, ON995616, ON995617, ON996162, OP996163, OP082332, OP082333, OP082334, OP082335, OP082336, OP082337 and OP101175). On the NCBI-BLAST analysis, they were closely related to *M. haemocanis* (> 99% identity with 100% query coverage). They clustered with *M. haemocanis* from other countries and formed a single clade along with the *M. haemofelis* isolates from cats (Figure 1). The mean group genetic distance among *M. haemocanis and M. haemofelis* isolates based on the *16S rDNA* sequences was negligible (GD = 0.002 3) (0.2%).

**Figure 2:**
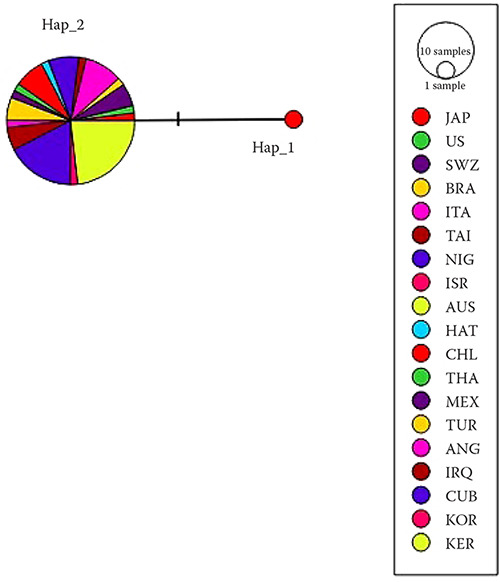
The median joining network of the *M. haemocanis* species determined by using 53 *16S rRNA* sequences

The haplotype analysis of *M. haemocanis* using the *16S rDNA* identified two haplotypes in accordance with the variable site distribution. Haplotype (H1) was shown by the *M. haemocanis* isolate from Japan (GenBank: AB848714). Haplotype 2 (H2) showed the highest frequency. The majority of the isolates of *M. haemocanis* (*n* = 53) from different countries including the isolates from south India were clustered in H2 (Figure 2).

## DISCUSSION

There are two haemoplasmas (*M. haemocanis* and *Candidatus* Mycoplasma haematoparvum) reported from both wild and domestic canines in the world (Baker and Tasker 2016; Altay et al. 2020; Ufuk et al. 2021). The present study confirmed the prevalence of one species of canine haemoplasma, viz., *M. haemocanis* in the dogs of Kerala, a south Indian state. Microscopic examination did not detect any *Mycoplasma* organisms in the blood smears of the apparently healthy community owned dogs in the present study. However, a prevalence of 18.6% was observed for *M. haemocanis* in these dogs when a PCR analysis was employed. Earlier, an *M. haemocanis* prevalence of 2.75% by microscopy in dogs from Kannur, north Kerala (Preena et al. 2020) and 12.2% in north Indian states by PCR (Abd Rani et al. 2011) was recorded.

The phylogenetic tree constructed in the present study based on the partial *16S rRNA* gene sequences clustered *M. haemocanis* and *M. haemofelis* in a single clade (Tasker et al. 2003; Roblejo-Arias et al. 2022). The phylogenetic tree did not revealed any obvious geographical or host specificity grouping of the *M. haemocanis* sequences (Tasker et al. 2003; Aquino et al. 2016). The partial *16S rRNA* gene of the *M. haemocanis* sequences generated in our study showed no genetic variability when compared with other *M. haemocanis* isolates from dogs in different countries (Tasker et al. 2003; Novacco et al. 2010; Aquino et al. 2016) (Figure 1). It was further supported by the genetic distance analysis.

The phylogenetic analysis positioned the haemoplasma species into two distinct groups, one clade comprising *M. haemocanis* and *M. haemofelis*, while the other consisting of *Candidatus M*. *haemolamae*, *M.* *wenyonii*, *M.* *ovis*, *M.* *suis*, *Candidatus M*. *haematoparvum* and *Candidatus M*. *haemominutum* (Tasker et al. 2003; Ramos et al. 2021; Roblejo-Arias et al. 2022). The haplotype analysis also supported the absence of variability among the Indian isolates when compared with the other *M. haemocanis* isolates from dogs of different countries (Figure 2).

The high prevalence (18.6%) of *M. haemocanis* in the community owned dogs in the present study may be attributed to the arthropod vectors prevalent in the area including *R. sanguineus*, which could also be able to parasitise wild fauna such as rodents, canids, felids and swine (Andre et al. 2011). The biting and fighting habits in dogs and the mechanical transmission by fresh blood-contaminated fomites or by blood-sucking arthropods play a major role in the transmission cycle of *M. haemocanis* infections from community owned dogs to the domestic population (Messick 2004; Sasaki et al. 2008; Willi et al. 2010; Baker and Tasker 2016).

The *RNase P*, *RNA* (*rnpB*) gene and non-ribosomal genes, such as *gapA* and *dnaK*, are proposed as other suitable markers (do Nascimento et al. 2012; Zheng et al. 2017; Roblejo-Arias et al. 2022) that could be used for establishing the phylogenetic relationship of *Mycoplasma* organisms from dogs and cats.

Further characterisation using these genes for the *Mycoplasma* organisms may reveal their genetic variations, if present. Since the organisms are prevalent in the south Indian canine population, the diagnosis of *Mycoplasma* infections should be the part of differential diagnosis for haemolytic anaemia in susceptible dog populations. Presently, antibiotics like oxytetracycline and doxycyclin can be used for therapy of *Mycoplasma* infections in dogs (Maes et al. 2020). Vaccines against these organisms are not available in the country at present. In addition, the prevalence of the *Mycoplasma* infections in dogs necessitates proper tick control strategies for canine populations.

The present study confirmed presence of *M. haemocanis* in the community owned dogs of Kerala, south India. The clinical relevance of haemotropic *Mycoplasma* infections in canines needs to be understood.

## Conflict of interest

The authors declare no conflict of interest.
